# Lactate and Immunosuppression in Sepsis

**DOI:** 10.1097/SHK.0000000000000958

**Published:** 2018-01-12

**Authors:** Benjamin Nolt, Fei Tu, Xiaohui Wang, Tuanzhu Ha, Randi Winter, David L. Williams, Chuanfu Li

**Affiliations:** ∗ Department of Surgery, James H. Quillen College of Medicine, East Tennessee State University, Johnson City, Tennessee; † Center of Excellence in Inflammation, Infectious Disease and Immunity, James H. Quillen College of Medicine, East Tennessee State University, Johnson City, Tennessee

**Keywords:** Aerobic glycolysis, immunosuppression, lactate, sepsis/septic shock

## Abstract

Serum lactate levels are traditionally interpreted as a marker of tissue hypoxia and often used clinically as an indicator of severity and outcome of sepsis/septic shock. Interestingly, recent studies involving the effects of tumor-derived lactate suggest that lactate itself may have an immunosuppressive effect in its local environment. This finding adds to the recent advances in immunometabolism that shed light on the importance of metabolism and metabolic intermediates in the regulation of innate immune and inflammatory responses in sepsis. In this article, we summarize recent studies, showing that the activation of immune cells requires aerobic glycolytic metabolism and that lactate produced by aerobic glycolysis may play an immunosuppressive role in sepsis.

## INTRODUCTION

Sepsis is a clinical syndrome characterized by systemic inflammatory response to infection (1–3). In the early stage of sepsis, activated innate immune cells initiate a significant increase in both innate immune and inflammatory responses to clear invading pathogens from the host. If the initial response is not properly controlled, it will result in exaggerated innate immune and inflammatory responses that could damage organs (2, 4) and increase septic mortality (1). Early clinical efforts are focused on controlling the inflammatory responsive phase of sepsis. Unfortunately, over the last 20 years unanimously poor results have been obtained from clinical trials using anti-inflammatory targets (5). Recent data show that the mortality of sepsis has been significantly reduced due to improvements in the treatment protocols (6). Furthermore, those who survive from the acute hyperinflammatory responsive phase remain at an increased risk for secondary and/or nosocomial infection, and consequential late-stage mortality (7, 8). However, the mechanisms are incompletely understood. Hotchkiss, Payen, and Pickkers as well as others recently hypothesized that an immunosuppressive phase may exist in sepsis/septic patients (3, 9–15) and may alternatively be a better candidate for therapy (16, 17). Although this hypothesis seems controversial (18, 19), there are number of factors that contribute to immunosuppression, including the apoptosis of innate immune cells and most T-cell populations (9), a decrease in the number of lymphoid progenitors (20), a reduction in bone marrow cell production (21), and a resultant state of immune tolerance/paralysis in which many immune cells are reprogrammed via epigenetic alterations to an unresponsive phenotype (22). In addition, metabolic reprogramming of immune cells may also contribute to the development of immune dysfunction during sepsis (23). Importantly, increasing evidence shows that extracellular lactate may have an important regulatory effect on a variety of immune cells (24). In addition, excellent review articles have been published showing that aerobic glycolytic metabolism is necessary for the activation of immune cells (23, 25–28). In this article, we briefly discuss lactate's potential inhibitory role in the systemic immune response to sepsis.

## SERUM LACTATE LEVELS IN SEPSIS

The measurement of serum lactate levels is often incorporated in the clinical management of critical illness, particularly in cases of severe sepsis and septic shock (29). In this context, serum lactate is typically used to evaluate disease severity, treatment response, and prognosis (30). The recent sepsis-3 guidelines recommend that persistence of a serum lactate more than 2 mmol/L, despite adequate fluid resuscitation, should be included as a new criterion when clinically defining septic shock (31). This recommendation is based on the recognition that lactate levels correlate strongly and positively with disease severity, morbidity, and mortality in the context of sepsis (32, 33). Published literature has shown that high concentrations of serum lactate could be a predictor of mortality, whereas reduced lactate levels have been reported to be associated with improved clinical outcomes (15, 29, 34–38). Vincent et al. (39) recently published an excellent review article regarding the value of blood lactate kinetics in critically ill patients. The authors systematically searched the published literatures, collected data from 96 studies, and concluded that a better outcome was associated with decreasing blood lactate concentrations. This was not limited to septic patients, suggesting that the value of lactate kinetics seems to be valid regardless of the initial value (39). The authors preferred using the term lactate kinetics that reflect the greater lactate production than clearance (39). Indeed, recent studies highlight the important role of immune cells in the production of lactate through aerobic glycolytic metabolism (25, 26, 40, 41).

## INCREASED LACTATE PRODUCTION BY ACTIVATED IMMUNE CELLS THROUGH AEROBIC GLYCOLYSIS

Over half a century ago, Dr. Otto Warburg observed that various cancer cells metabolize glucose directly to lactate despite the presence of abundant oxygen in the environment (42). Initially termed the Warburg effect, this phenomenon is now more commonly referred to as aerobic glycolysis. Recently, the transient utilization of aerobic glycolysis has been observed in many activated immune cells (43, 44). Although it is significantly less favorable energetically, there are several advantages to this type of metabolism with regard to immune function. First, an adequate immune response requires rapid energy production, and aerobic glycolysis provides the essential ATP immediately (45). Second, aerobic glycolysis and parallel increases in the pentose phosphate pathway provide important precursors for the synthesis of lipids, amino acids, and nucleotides that are required for rapid cellular growth and proliferation (25). In addition to supporting the basic energy requirements of the dividing cell, altered metabolism is now known to play an important and direct role in regulating changes in immune cell phenotype. For example, recent studies have shown that metabolic enzymes and their products can stimulate the release of alarmins from cells (46), act as bacterial component receptors (47), promote epigenetic modification of histones for trained immunity (48), regulate microRNA expression (49), and participate in various other immunoregulatory processes. Collectively, these findings suggest that the transient adoption of aerobic glycolysis in the immune response may also play an active role in regulating cell phenotype (50).

Indeed, the switch to aerobic glycolysis seems to play an important role in the inflammatory response by the innate immune system. Toll-like receptors (TLRs) are critical in the induction of innate immune and inflammatory responses. TLRs could bind with their ligands, including bacterial components and endogenous ligands, and initiate a cellular signaling cascade that ultimately results in transcriptional regulatory changes within the cell (51, 52). For example, lipopolysaccharides (LPS) binds with TLR4 on dendritic cells (DCs) and macrophages, leading to a metabolic transition from oxidative phosphorylation to aerobic glycolysis and resulting in a proinflammatory phenotype (53, 54, 40). Suzuki et al. recently outlined the differential reliance of proinflammatory (M1) macrophages on aerobic glycolysis. This is in contrast to alternative (M2) phenotypes that rely more heavily on oxidative phosphorylation (55). In addition to these observations, *in vitro* inhibition of glycolysis seems to reprogram innate immune cells to a more anti-inflammatory state, further highlighting the importance of metabolism in cell phenotype (56, 57).

In addition, adaptive immunity also plays an important role in mediating the pathogen-specific and delayed response in sepsis. The metabolic regulation of adaptive immune cells has been reported in subsets of both B and T lymphocytes that adopt a Warburg-like metabolism upon activation (58–60). In lymphocytes, metabolic regulation seems to be different between T-effector (T_eff_) cells and T-regulatory (T_reg_) cells. Michalek et al. recently reported that T_eff_ cells exhibit greater expression of the glucose transporter 1 (GLUT1) and elevated levels of glycolysis, while T_reg_ cells primarily rely on fatty acid oxidation (61). This finding may highlight a characteristic metabolic difference between predominantly proinflammatory cells. For example, T_eff_ and M1 macrophage subtypes rely primarily on glycolysis, whereas T_reg_ and M2 macrophage subtypes use fatty acid oxidation. Ultimately, these variations may represent an important feature by which immune cells can respond differently to metabolites present in their surroundings.

## DECREASED PRODUCTION OF LACTATE IMPROVES SURVIVAL OUTCOME OF SEPTIC MICE

Critical illness usually causes a metabolic shift from mitochondrial oxidative phosphorylation to aerobic glycolysis. This transition is associated with lactate production, multiple organ dysfunction, and poor outcomes. As previously mentioned, utilization of aerobic glycolytic metabolism by activated immune cells could contribute to increase lactate production. Nalos et al. used transcriptomic analysis to examine the cellular metabolism of circulating blood cells from nonhypoxic critically ill patients and observed a significant reprogramming of metabolic pathways during critical illness. These authors concluded that aerobic glycolysis does exist in nonhypoxic cells during critical illness (62). The increased lactate production may also indicate a metabolic shift to an inflammatory glycolysis. Palsson-McDermott et al. have shown that stimulation of macrophages with LPS significantly increased the expression of pyruvate kinase M2 (PKM2), a critical modulator of IL-1β production, macrophage polarization, glycolytic reprogramming, and Warburg metabolism (63). Furthermore, activation of PKM2 attenuated LPS-induced proinflammatory M1 macrophage phenotype and promoted traits typical of an M2 macrophage (63). Xie et al. (64) from the same group reported that PKM2-mediated glycolysis promotes inflammasome activation by modulating EIF1AK2 phosphorylation in macrophages. In accordance with these findings, pharmacological inhibition of the PKM2-EIF2AK2 pathway has been shown to protect mice from lethal endotoxemia and polymicrobial sepsis (64). Inhibition of aerobic glycolysis by either 2-deoxy-d-glycose (2-DG) or PKM2 inhibitor also markedly improves survival outcome in polymicrobial sepsis, and reduces serum lactate levels and HMGB1 release (46). Wang et al. reported a similar observation that inhibition of aerobic glycolysis by 2-DG significantly improved survival outcome in bacterial sepsis (65) and reduced LPS-induced inflammation *in vivo*(66). Recently, Zheng et al. (67) reported that sepsis-increased glycolysis also contributes to cardiomyopathy and mortality. Inhibition of glycolysis by 2-DG markedly improves cardiac function and survival outcome by improving mitochondrial function and inflammatory responses (67). Collectively, the current published literature indicates that sepsis and endotoxin could increase aerobic metabolism and produce more lactate that may ultimately alter the function of immune cells. Understanding the mechanisms by which metabolic switching regulates the processes of immune response could be a novel research topic in sepsis.

## LACTATE MODULATES THE IMMUNE RESPONSE

Previous studies have shown that high levels of lactate could downregulate the rate-limiting glycolytic enzymes hexokinase and phosphofructokinase in a variety of tissues (68) and immune cells (69). Therefore, given the importance of aerobic glycolysis in activated immune cells, the downregulation of these rate-limiting glycolytic enzymes may have important implications on cellular function. Indeed, a growing body of evidence suggests that tumor-derived lactate has clinically relevant immunosuppressive effects on a variety of cell types in the surrounding microenvironment (70). Interestingly, in cancer research, the immunologic changes of immune cells are very similar to those observed in the immunosuppressive phase of sepsis. It would be interesting to investigate the effect of increased lactate on immune cell function during sepsis.

## LACTATE AND INNATE IMMUNE CELL FUNCTION

Recent and ongoing studies have explored the potential immunomodulatory effects of lactate on innate immune cells, primarily macrophages and DCs. These cells serve a fundamental role as antigen-presenting cells, and act as gatekeepers for the activation of lymphocyte B and T cells in the adaptive response. In the context of sepsis, impaired function of innate immunity not only limits the response to primary infection, but also damages important barriers to secondary nosocomial infection (17). A prominent feature of protracted sepsis is the inappropriate development of immunologic tolerance toward pathogens (9, 71). For example, DCs transition toward a progressively tolerogenic phenotype and promote immunosuppressive regulatory T-cell differentiation (72). The mechanisms underlying this transition may involve a significant metabolic dysfunction within these cells (23, 73). Indeed, recent studies have reported that the addition of exogenous lactate to growth medium containing DCs induced metabolic reprogramming and ultimately triggered innate immune cells to adapt a more tolerogenic phenotype (73, 74). These authors proposed that the unfavorable concentration gradient of lactate may prevent its diffusion-mediated export from immunogenic DCs that rely on aerobic glycolysis. It has also been reported that lactate in peritoneal dialysis solutions may inhibit LPS-induced maturation of DCs (75).

Recently, the effects of lactate on the functioning and differentiation of macrophages have been reported (56, 76, 77). In the late stage of sepsis, macrophages are often observed as having a predominantly immunosuppressive M2 phenotype configuration that may have a critical role in the pathogenesis of immune system dysfunction (56, 77). Interestingly, the M2 phenotype has also been observed in the local environments of tumor cells, where it may contribute to immune system evasion (76). Colegio et al. (76) reported that lactate may serve as the primary mediator responsible for promoting the M2 inhibitory polarization of macrophages. In subsequent *in vitro* experiments involving bone marrow-derived macrophages, these authors reported that lactate was consistently capable of inducing an M2-like macrophage polarization by an HIF-1α-dependent mechanism. In addition, lactate treatment also increased production of M2-associated genes (VEGF and Arg1) and markers (Fizz1, Mgl1, and Mgl2) in a dosage-dependent manner (76). Selleri et al. (78) have similarly reported that lactate induces a preferential differentiation of monocytes into M2 macrophages in a dose-dependent fashion by metabolic reprogramming. Furthermore, it has been reported that lactate decreases TNF-α secretion by human monocytes (79), potentially by reducing NF-κB activation and delaying LPS-induced signal transduction (80).

**Fig. 1:**
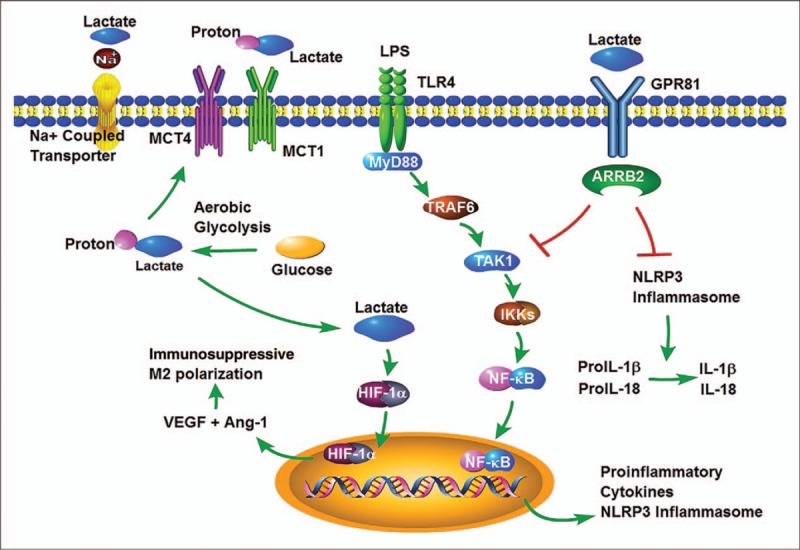
Possible mechanisms by which lactate can induce the macrophage transition to an anti-inflammatory phenotype.

To explore the mechanisms by which lactate can induce the macrophage transition to an anti-inflammatory phenotype, Hoque et al. (81) recently proposed a novel cellular signaling pathway. This pathway involves the GPR81 receptor that recognizes lactate and has the ability to induce the transition of macrophages to the M2 phenotype. As presented in Figure 1, these authors showed that macrophages treated with LPS in the presence of lactate exhibited a significant reduction in proinflammatory cytokine production such as Pro-IL1 β, Pro-IL18, Casp1, and Nlrp3, whereas production of anti-inflammatory cytokines like IL-10 was not affected. The mechanisms by which lactate significantly affects LPS-induced production of proinflammatory cytokines involve the GPR81-dependent antagonism of the TLR4/TLR9-mediated signaling pathway, and consequently attenuation of LPS-induced NF-κB activation. It has been reported that GPR81 has an impressively high affinity for lactate with an estimated EC50 of ∼4.3 mM/L (82), suggesting that relatively low concentrations of lactate may have an effect on TLR-mediated NF-κB activation pathway (81).

Lactate has also been reported to influence the bone marrow stem cell maturation process. Husain et al. have recently shown that the addition of lactate to the growth medium before induction of bone marrow stem cell differentiation results in a significantly increased production of myeloid-derived suppressor cells (MDSCs) when compared with the control (83). MDSCs are a heterogenous group of cells that have predominantly immunosuppressive effects and play an important role in cancer development and chronic infectious diseases (84). In addition, increased MDSCs in sepsis were recently implicated in the pathogenesis of protracted immune dysfunction (20, 85). However, to date few studies have neither examined the metabolic properties of these cells nor fully described the mechanisms surrounding their origin.

## LACTATE AND ADAPTIVE IMMUNE CELL FUNCTION

In addition to influencing the function of the innate immune cells, lactate has also been reported to have effects on T-cell functioning (86). Haas et al. (69) recently reported that lactate accumulation in the synovia of rheumatoid arthritis patients may play a role in the localization of T cells to the site of inflammation. *In vitro* studies by this group demonstrated that sodium lactate inhibited CD4+ cell motility, whereas an acidic lactate was required to inhibit the motility of CD8+ T cells. In CD4+ T cells, the effect of lactate (at physiologic pH) seemed to be dependent on the interruption of glycolysis and required expression of the Na+/lactate cotransporter, Slc5a12 (69). In contrast, the inhibitory effect of lactate on CD8+ T cells required expression of the Slc16a1 proton-coupled lactate transporter in addition to an acidic microenvironment (69). These effects were dose-dependent and estimated the EC50 of lactate to be ∼10 mM/L (69). However, this concentration is not significantly greater than what is observed in severe sepsis. Finally, the authors reported that buffered sodium lactate induced CD4^+^ T helper cells toward a TH17 subset, whereas unbuffered lactic acid inhibited the cytolytic function of CD8^+^ T cells (69). At present, there is no study to investigate the effects of lactate on T_reg_ cells. It is possible that the proposed T_reg_ cells are less dependent on aerobic glycolysis and primarily use oxidative phosphorylation for their energy production (61). Thus, theoretically they may not be susceptible to this type of metabolic regulation.

## CONCLUSION

**Fig. 2:**
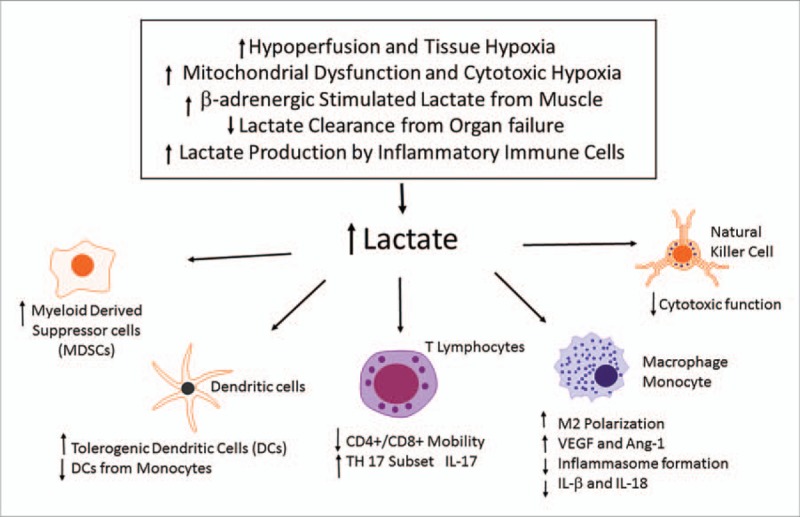
Potential role of increased lactate in the regulation of immune cell function.

We have highlighted some of the current research surrounding the potential role of lactate as an immunosuppressive metabolite (see Fig. 2). Although there are clearly a number of contributing factors to the development of immune suppression in sepsis, the recent developments in other fields of research suggest that lactate could be a potential and critical contributory factor in the regulation of immune function in sepsis.
